# Supplementary material for: Roles of C/EBP class bZip proteins in the growth and cell competition of Rp (‘Minute’) mutants in Drosophila
Source: eLife. 2020 Jan 7;9:e50535. doi: 10.7554/eLife.50535 (PMC6946401; doi:10.7554/eLife.50535)
Supplement: Supplementary file 1. [file elife-50535-supp1.docx]

| **Key Resources Table** | | | | |
| --- | --- | --- | --- | --- |
| **Reagent type (species) or resource** | **Designation** | **Source or reference** | **Identifiers** | **Additional information** |
| gene (*Drosophila melanogaster*) | Xrp1 |  | FLYBASE:FBgn0261113 |  |
| gene (*Drosophila melanogaster*) | Irbp18 |  | FLYBASE:FBgn0036126 |  |
| gene (*Drosophila melanogaster*) | dATF4/Crc |  | FLYBASE:FBgn0000370 |  |
| genetic reagent (*D. melanogaster*) | Xrp1^m2-73^ | (Lee et al., 2016) | FLYBASE:FBal0346068 | Bloomington Drosophila Stock Center #81270 |
| genetic reagent (*D. melanogaster*) | Xrp1^02515^ | (Spradling et al., 1999) | FLYBASE: FBal0009448 | Bloomington Drosophila Stock Center #11569 |
| genetic reagent (*D. melanogaster*) | Xrp1^attP^ ^flox^ | this study |  | See Figure 4A; Strain maintained in Dr. Nicholas Baker’s lab. |
| genetic reagent (*D. melanogaster*) | Xrp1^attP^ ^loxP^ | this study |  | See Figure 4A; Strain maintained in Dr. Nicholas Baker’s lab. |
| genetic reagent (*D. melanogaster*) | Xrp1^HA^ | this study |  | See Figure 4A; Strain maintained in Dr. Nicholas Baker’s lab. |
| genetic reagent (*D. melanogaster*) | Xrp1^DAT-HA^ | this study |  | See Figure 8C; Strain maintained in Dr. Nicholas Baker’s lab. |
| genetic reagent (*D. melanogaster*) | Xrp1^DBR-HA^ | this study |  | See Figure 8C; Strain maintained in Dr. Nicholas Baker’s lab. |
| genetic reagent (*D. melanogaster*) | UAS-Xrp1 | (Tsurui-Nishimura et al., 2013) | FLYBASE:FBal0284387 |  |
| genetic reagent (*D. melanogaster*) | UAS-Xrp1^HA^ | this study |  | See Figure 8C; Strain maintained in Dr. Nicholas Baker’s lab. |
| genetic reagent (*D. melanogaster*) | UAS-Xrp1^DAT-HA^ | this study |  | See Figure 8C; Strain maintained in Dr. Nicholas Baker’s lab. |
| genetic reagent (*D. melanogaster*) | UAS-Xrp1^DBR-HA^ | this study |  | See Figure 8C; Strain maintained in Dr. Nicholas Baker’s lab. |
| genetic reagent (*D. melanogaster*) | UAS-Xrp1^DLZ-HA^ | this study |  | See Figure 8C; Strain maintained in Dr. Nicholas Baker’s lab. |
| genetic reagent (*D. melanogaster*) | UAS-  Xrp1^1-546^ | this study |  | See Materials and Methods; Strain maintained in Dr. Nicholas Baker’s lab. |
| genetic reagent (*D. melanogaster*) | UAS-Irbp18 | this study |  | See Materials and Methods; Strain maintained in Dr. Nicholas Baker’s lab. |
| genetic reagent (*D. melanogaster*) | UAS-hDDIT3 | this study |  | See Materials and Methods; Strain maintained in Dr. Nicholas Baker’s lab. |
| genetic reagent (*D. melanogaster*) | Irbp18^05006^ | (Francis et al., 2016) | FLYBASE: FBal0185260 | Exelisis Drosophila Collection  #f05006 |
| genetic reagent (*D. melanogaster*) | UAS-p35 | (Neufeld et al., 1998) | FLYBASE:FBti0012595 | Bloomington Drosophila Stock Center #5073 |
| genetic reagent (*D. melanogaster*) | UAS-diap1 | FLYBASE:[FBrf0173262](http://flybase.org/reports/FBrf0173262) | FLYBASE:FBti0038641 | Bloomington Drosophila Stock Center  #6657 |
| genetic reagent (*D. melanogaster*) | UAS-yki | (Huang et al., 2005) | FLYBASE:FBal0191232 |  |
| genetic reagent (*D. melanogaster*) | Rheb^AV4^  (UAS-Rheb) | (Patel et al., 2003) | FLYBASE:FBal0141561 | Bloomington Drosophila Stock Center  #9690 |
| genetic reagent (*D. melanogaster*) | UAS-dsRNA^Xrp1^ | (Ni et al., 2011) | FLYBASE: FBti0144696 | Bloomington Drosophila Stock Center  #34521 |
| genetic reagent (*D. melanogaster*) | UAS-dsRNA^irbp18^ | (Ni et al., 2011) | FLYBASE: FBti0140125 | Bloomington Drosophila Stock Center  #33652 |
| genetic reagent (*D. melanogaster*) | UAS-dsRNA^w^ | (Ni et al., 2011) | FLYBASE:FBtp0064645 | Bloomington Drosophila Stock Center  #33623 |
| genetic reagent (*D. melanogaster*) | UAS-dsRNA^crc^ | (Dietzl et al., 2007) | FLYBASE:FBst0457929 | Vienna Drosophila Resource Center #v2934 |
| genetic reagent (*D. melanogaster*) | UAS-dsRNA^crc^ | (Dietzl et al., 2007) | FLYBASE:FBst0457935 | Vienna Drosophila Resource Center #v2935 |
| genetic reagent (*D. melanogaster*) | Df(3L)H99 | (Abbott and Lengyel, 1991) | FLYBASE:FBab0022359 | Bloomington Drosophila Stock Center  #1576 |
| genetic reagent (*D. melanogaster*) | UAS-dsRNA^dronc^ | (Dietzl et al., 2007) | FLYBASE:FBti0118486 | Vienna Drosophila Resource Center #v100424 |
| genetic reagent (*D. melanogaster*) | UAS-dsRNA^Strica^ | (Dietzl et al., 2007) | FLYBASE:FBst0454607 | Vienna Drosophila Resource Center #v22594 |
| genetic reagent (*D. melanogaster*) | UAS-dsRNA^dark^ | (Ni et al., 2011) | FLYBASE:FBal0257547 |  |
| genetic reagent (*D. melanogaster*) | UAS-dsRNA^dredd^ | (Ni et al., 2011) | FLYBASE:FBst0457259 | Vienna Drosophila Resource Center #v28041 |
| genetic reagent (*D. melanogaster*) | arm-LacZ | (Vincent et al., 1994) | FLYBASE:FBal0040819 |  |
| genetic reagent (*D. melanogaster*) | Ubi-GFP | (Davis et al., 1995) | FLYBASE:FBal0047085 |  |
| genetic reagent (*D. melanogaster*) | Df(1)su(s)R194 | (Duffy et al., 1996) | FLYBASE:FBab0024817 | Deletes *RpL36* |
| genetic reagent (*D. melanogaster*) | P[RpL36+ w+] | (Tyler et al., 2007) | FLYBASE: FBal0193398 |  |
| genetic reagent (*D. melanogaster*) | M(2)56F  (mutating RpS18) | Laboratory of Y. Hiromi | FLYBASE:FBal0011916 |  |
| genetic reagent (*D. melanogaster*) | hs-FLP | (Struhl and Basler, 1993) | FLYBASE:FBtp0001101 |  |
| genetic reagent (*D. melanogaster*) | hs-Cre | (Siegal and Hartl, 1996) | FLYBASE:FBti0012692 | Bloomington Drosophila Stock Center  #851 |
| genetic reagent (*D. melanogaster*) | hs-I-SceI | FLYBASE:[FBrf0157298](http://flybase.org/reports/FBrf0157298) | FLYBASE:FBti0026981 | Bloomington Drosophila Stock Center  #25679 |
| genetic reagent (*D. melanogaster*) | ey-FLP | (Newsome et al., 2000) | FLYBASE:FBal0098303 |  |
| genetic reagent (*D. melanogaster*) | M{vas-int.Dm}  ZH-2A | (Bischof et al., 2007) | FLYBASE:FBti0099694 | Bloomington Drosophila Stock Center  #40161 |
| genetic reagent (*D. melanogaster*) | Ubi-gal4 | (Baena-Lopez et al., 2013) | FLYBASE:FBtp0094799 |  |
| genetic reagent (*D. melanogaster*) | tubP-GAL4 | (Lee and Luo, 1999) | FLYBASE:FBal0097158 |  |
| genetic reagent (*D. melanogaster*) | GMR-gal4 | (Freeman, 1996) | FLYBASE:FBtp0001315 |  |
| genetic reagent (*D. melanogaster*) | UAS-mCD8::  GFP | (Lee and Luo, 1999) | FLYBASE:FBal0097215 | Bloomington Drosophila Stock Center  #5137 |
| genetic reagent (*D. melanogaster*) | tubP-GAL80 | (Lee and Luo, 1999) | FLYBASE:FBal0097120 | Bloomington Drosophila Stock Center  #5135, 5191 |
| genetic reagent (*D. melanogaster*) | P{GAL4-Act5C(FRT.CD2).P}S | (Pignoni and Zipursky, 1997) | FLYBASE:FBti0012408 | Bloomington Drosophila Stock Center  #51308 |
| genetic reagent (*D. melanogaster*) | P{neoFRT}42D | (Xu and Rubin, 1993) | FLYBASE:FBti0141188 | Bloomington Drosophila Stock Center  #1802 |
| genetic reagent (*D. melanogaster*) | P{neoFRT}80B | (Xu and Rubin, 1993) | FLYBASE:FBti0002073 | Bloomington Drosophila Stock Center  #1988 |
| genetic reagent (*D. melanogaster*) | P{neoFRT}82B | (Xu and Rubin, 1993) | FLYBASE:FBti0002074 | Bloomington Drosophila Stock Center #2050, 2051 |
| genetic reagent (*D. melanogaster*) | P{neoFRT}82B | (Xu and Rubin, 1993) | FLYBASE:FBti0002074 | Bloomington Drosophila Stock Center #2050, 2051 |
| genetic reagent (*D. melanogaster*) | w[*]; P{ry[+t7.2]=Act5C(FRT.polyA)lacZ.nls1}2, P{w[+mC]=Ubi-p63E(FRT.STOP)Stinger}9F6/CyO; P{w[+mC]=GAL4-Act5C(FRT.CD2).P}S, P{w[+mC]=UAS-His-RFP}3/TM3, Sb[1] | (Worley et al., 2013) | FLYBASE: FBst0051308 | Bloomington Drosophila Stock Center #51308 |
| Antibody | polyclonalRabbit anti-XRP1(short) | (Francis et al., 2016) |  | 1:200 dilution |
| Antibody | polyclonalRabbit anti-IRBP18 | (Francis et al., 2016) |  | 1:100 dilution |
| Antibody | Mouse anti-HA  Tag monoclonal | Cell Signalling Technology | Cat #2367 | 1:100 dilution |
| Antibody | Mouse anti-beta galactosidase (mAb40-1a) monoclonal | DSHB | RRID: [AB_2314509](http://antibodyregistry.org/AB_2314509) | 1:100 dilution |
| Antibody | Rabbit anti-active-Dcp1 polyclonal | Cell Signalling Technology | Cat #9578 | 1:50 dilution |
| Antibody | [Goat anti-Mouse IgG, Alexa Fluor 488](https://www.thermofisher.com/antibody/product/A32723)  polyclonal | Thermo Fischer Scientific | Cat #  A-11001 | 1:200 dilution |
| Antibody | [Goat anti-Rabbit IgG, Alexa Fluor 488](https://www.thermofisher.com/antibody/product/A32723)  polyclonal | Thermo Fischer Scientific | Cat # A-11008 | 1:200 dilution |
| Antibody | [Goat anti-Mouse IgG, Alexa Fluor 568](https://www.thermofisher.com/antibody/product/A32723)  polyclonal | Thermo Fischer Scientific | Cat #  A-11004 | 1:200 dilution |
| Antibody | [Goat anti-Rabbit IgG, Alexa Fluor 568](https://www.thermofisher.com/antibody/product/A32723)  polyclonal | Thermo Fischer Scientific | Cat #  A-11011 | 1:200 dilution |
| Antibody | [Goat anti-Mouse IgG, Alexa Fluor 647](https://www.thermofisher.com/antibody/product/A32723)  polyclonal | Thermo Fischer Scientific | Cat #  A-21235 | 1:200 dilution |
| Antibody | [Goat anti-Rabbit IgG, Alexa Fluor 647](https://www.thermofisher.com/antibody/product/A32723)  polyclonal | Thermo Fischer Scientific | Cat #  A-21244 | 1:200 dilution |
| recombinant DNA reagent reagent | pUAST vector | (Brand and Perrimon, 1993) | FLYBASE:FBmc0000383 | Drosophila Genomics Resource Center  #1000 |
| recombinant DNA reagent | pUAST-irbp18 | this study |  | See Materials and Methods; Dr. Nicholas Baker’s lab. |
| recombinant DNA reagent | pUAST-hDDIT3 | this study |  | See Materials and Methods; Dr. Nicholas Baker’s lab. |
| recombinant DNA reagent | pUAST-Xrp1^HA^ | this study |  | See Materials and Methods; Dr. Nicholas Baker’s lab. |
| recombinant DNA reagent | pUAST-Xrp1^DAT-HA^ | this study |  | See Materials and Methods; Dr. Nicholas Baker’s lab. |
| recombinant DNA reagent | pUAST-Xrp1^DBS-HA^ | this study |  | See Materials and Methods; Dr. Nicholas Baker’s lab. |
| recombinant DNA reagent | pUAST- Xrp1^DLZ-HA^ | this study |  | See Materials and Methods; Dr. Nicholas Baker’s lab. |
| recombinant DNA reagent | pUAST- Xrp1^1-546^ | this study |  | See Materials and Methods; Dr. Nicholas Baker’s lab. |
| recombinant DNA reagent | pTVCherry  vector | (Baena-Lopez et al., 2013) |  |  |
| recombinant DNA reagent | pTV-Xrp1 | this study |  | See Materials and Methods; Dr. Nicholas Baker’s lab. |
| recombinant DNA reagent | RIVFRT MCS FRT MCS3  vector | (Baena-Lopez et al., 2013) |  |  |
| recombinant DNA reagent | pRIVW | this study |  | See Materials and Methods; Dr. Nicholas Baker’s lab. |
| recombinant DNA reagent | pRIVW- Xrp1^HA^ | this study |  | See Materials and Methods; Dr. Nicholas Baker’s lab. |
| recombinant DNA reagent | pRIVW- Xrp1^DAT-HA^ | this study |  | See Materials and Methods; Dr. Nicholas Baker’s lab. |
| recombinant DNA reagent | pRIVW- Xrp1^DBS-HA^ | this study |  | See Materials and Methods; Dr. Nicholas Baker’s lab. |
| recombinant DNA reagent | pRIVW- Xrp1^DLZ-HA^ | this study |  | See Materials and Methods; Dr. Nicholas Baker’s lab. |

Abbott, M.K., and Lengyel, J.A. (1991). Embryonic head involution and rotation of male terminalia require the Drosophila locus head involution defective. Genetics *129*, 783-789.

Baena-Lopez, L.A., Alexandre, C., Mitchell, A., Pasakarnis, L., and Vincent, J.P. (2013). Accelerated homologous recombination and subsequent genome modification in Drosophila. Development *140*, 4818-4825.

Bischof, J., Maeda, R.K., Hediger, M., Karch, F., and Basler, K. (2007). An optimized transgenesis system for Drosophila using germ-line-specific phiC31 integrases. Proc Natl Acad Sci U S A *104*, 3312-3317.

Brand, A.H., and Perrimon, N. (1993). Targeted gene expression as a means of altering cell fates and generating dominant phenotypes. Development *118*, 401-415.

Davis, I., Girdham, C.H., and O'Farrell, P.H. (1995). A nuclear GFP that marks nuclei in living Drosophila embryos; maternal supply overcomes a delay in the appearance of zygotic fluorescence. Dev Biol *170*, 726-729.

Dietzl, G., Chen, D., Schnorrer, F., Su, K.C., Barinova, Y., Fellner, M., Gasser, B., Kinsey, K., Oppel, S., Scheiblauer, S.*, et al.* (2007). A genome-wide transgenic RNAi library for conditional gene inactivation in Drosophila. Nature *448*, 151-156.

Duffy, J.B., Wells, J., and Gergen, J.P. (1996). Dosage-sensitive maternal modifiers of the drosophila segmentation gene runt. Genetics *142*, 839-852.

Francis, M.J., Roche, S., Cho, M.J., Beall, E., Min, B., Panganiban, R.P., and Rio, D.C. (2016). Drosophila IRBP bZIP heterodimer binds P-element DNA and affects hybrid dysgenesis. Proc Natl Acad Sci U S A *113*, 13003-13008.

Freeman, M. (1996). Reiterative use of the EGF receptor triggers differentiation of all cell types in the *Drosophila* eye. Cell *87*, 651-660.

Huang, J., Wu, S., Barrera, J., Matthews, K., and Pan, D. (2005). The Hippo signaling pathway coordinately regulates cell proliferation and apoptosis by inactivating Yorkie, the *Drosophila* homolog of YAP. Cell *122*, 421-434.

Lee, C.H., Rimesso, G., Reynolds, D.M., Cai, J., and Baker, N.E. (2016). Whole-Genome Sequencing and iPLEX MassARRAY Genotyping Map an EMS-Induced Mutation Affecting Cell Competition in Drosophila melanogaster. G3 (Bethesda) *6*, 3207-3217.

Lee, T., and Luo, L. (1999). Mosaic analysis with a repressible cell marker for studies of gene function in neuronal morphogenesis. Neuron *22*, 451-461.

Neufeld, T.P., de la Cruz, A.F., Johnston, L.A., and Edgar, B.A. (1998). Coordination of growth and cell division in the *Drosophila* wing. Cell *93*, 1183-1193.

Newsome, T.P., Asling, B., and Dickson, B.J. (2000). Analysis of *Drosophila* photoreceptor axon guidance in eye-specific mosaics. Development *127*, 851-860.

Ni, J.Q., Zhou, R., Czech, B., Liu, L.P., Holderbaum, L., Yang-Zhou, D., Shim, H.S., Tao, R., Handler, D., Karpowicz, P.*, et al.* (2011). A genome-scale shRNA resource for transgenic RNAi in Drosophila. Nat Methods *8*, 405-407.

Patel, P.H., Thapar, N., Guo, L., Martinez, M., Maris, J., Gau, C.L., Lengyel, J.A., and Tamanoi, F. (2003). Drosophila Rheb GTPase is required for cell cycle progression and cell growth. J Cell Sci *116*, 3601-3610.

Pignoni, F., and Zipursky, L. (1997). Induction of *Drosophila* eye development by Decapentaplegic. Development *124*, 271-278.

Siegal, M.L., and Hartl, D.L. (1996). Transgene Coplacement and high efficiency site-specific recombination with the Cre/loxP system in Drosophila. Genetics *144*, 715-726.

Spradling, A.C., Stern, D.F., Beaton, A., Rhem, E.J., Laverty, T., Mozden, N., Misra, S., and Rubin, G.M. (1999). The BDGP gene disruption project: sincle P element insertions mutating 25% of vital *Drosophila* genes. Genetics *153*, 135-177.

Struhl, G., and Basler, K. (1993). Organizing activity of *wingless* protein in *Drosophila*. Cell *72*, 527-540.

Tsurui-Nishimura, N., Nguyen, T.Q., Katsuyama, T., Minami, T., Furuhashi, H., Oshima, Y., and Kurata, S. (2013). Ectopic antenna induction by overexpression of CG17836/Xrp1 encoding an AT-hook DNA binding motif protein in Drosophila. Bioscience, biotechnology, and biochemistry *77*, 339-344.

Tyler, D.M., Li, W., Zhuo, N., Pellock, B., and Baker, N.E. (2007). Genes affecting cell competition in Drosophila. Genetics *175*, 643-657.

Vincent, J., Girdham, C., and O'Farrell, P. (1994). A cell-autonomous, ubiquitous marker for the analysis of *Drosophila* genetic mosaics. Developmental Biology *164*, 328-331.

Worley, M.I., Setiawan, L., and Hariharan, I.K. (2013). TIE-DYE: a combinatorial marking system to visualize and genetically manipulate clones during development in Drosophila melanogaster. Development *140*, 3275-3284.

Xu, T., and Rubin, G.M. (1993). Analysis of genetic mosaics in the developing and adult *Drosophila* tissues. Development *117*, 1223-1236.
